# MRI guided wire localization muscle biopsy in a child with juvenile dermatomyositis

**DOI:** 10.1186/1546-0096-11-15

**Published:** 2013-04-08

**Authors:** Victoria C Tuen, Shannon N Zingula, Christopher Moir, Ann M Reed, Jane M Matsumoto, David A Woodrum

**Affiliations:** 1 Pediatric Medicine, Mayo Clinic College of Medicine, Rochester, MN, 55905, USA; 2 Pediatric Radiology, Mayo Clinic College of Medicine, Rochester, MN, 55905, USA; 3 Children’s Center Surgery, Mayo Clinic College of Medicine, Rochester, MN, 55905, USA; 4 Pediatric Rheumatology, Mayo Clinic College of Medicine, Rochester, MN, 55905, USA; 5 Department of Interventional Radiology, Mayo Clinic College of Medicine, 200 First St. SW, Rochester, MN, 55905, USA

**Keywords:** Juvenile dermatomyositis, Myopathy, MRI, Guide wire localization

## Abstract

A novel technique for preoperative MRI guided wire localization for targeted surgical excisional biopsy of muscle is described in a pediatric patient with juvenile dermatomyositis (JDM). This technique allows for preoperative localization of abnormalities seen only with MRI. Using this technique, the patient underwent successful targeted muscle biopsy for confirmation of the diagnosis and staging of dermatomyositis.

## Background

Juvenile dermatomyositis (JDM) is an autoimmune, inflammatory myopathy. Dermatologic and muscle manifestations are most common, typically consisting of a heliotrope rash involving the eyelids, erythematous rash on the extensor surfaces (Gottron’s papules), and proximal muscle weakness [1,2]. Surgical muscle biopsy is diagnostic for cases in which the clinical presentation may not be clear. However, the muscle biopsy can be difficult because even within a single muscle group there can be heterogeneous inflammatory involvement. This patchy muscular involvement can only be delineated with magnetic resonance imaging (MRI) [3]. This complicates the surgical biopsy because grossly the muscular tissue all looks the same. Due to this, the surgeon must either take more tissue than is absolutely necessary or repeat biopsies may be required.

This case report presents a novel methodology for preoperative muscle localization in a patient needing surgical excisional biopsy for diagnosis of JDM. This technique utilizes MRI guidance for the advantage of soft tissue resolution and accurate targeting of the patchy muscular inflammation for surgical biopsy.

## Case presentation

A 5-year old boy presented to his family physician with a one week history of a raised, erythematous rash over the nasal bridge and ears. The patient had no additional symptoms. Initial diagnosis of atopic dermatitis versus allergy was made and treatment with mild topical steroid creams and antihistamines was initiated. Over the course of one month the rash worsened, spreading to involve the nose, bilateral cheeks and ears, and upper back. The patient’s mother noted worsening of the rash with sun exposure. Again, no other symptoms were noted at this time. Given the progressive nature of rash and failure to improve on topical steroids, the patient was referred to dermatology by his family physician.

Dermatologic evaluation described an erythematous, confluent papular rash on the bridge of the nose and extending over both cheeks, sparing the naso-labial folds. Several excoriations were noted in association with the rash. Similar rashes were present behind the ears and on the back, chest, and extensor surfaces of the upper and lower extremities bilaterally. Due to the characteristic malar rash and clinical presentation, differential diagnosis including JDM, cutaneous lupus, and atopic dermatitis were considered. A punch biopsy of the right posterior auricular region was performed which showed changes compatible with connective tissue disease such as lupus erythematosus or JDM including parakeratosis, epidermal atrophy, basal vacuolization, cytoid bodies along the dermal-epidermal junction, dermal telangiectasias and a moderate perivascular and perifollicular lymphocytic infiltrate in the dermis. The patient was treated with clobetasol topical steroid cream which resulted in mild improvement in the skin lesions.

**Figure 1:**
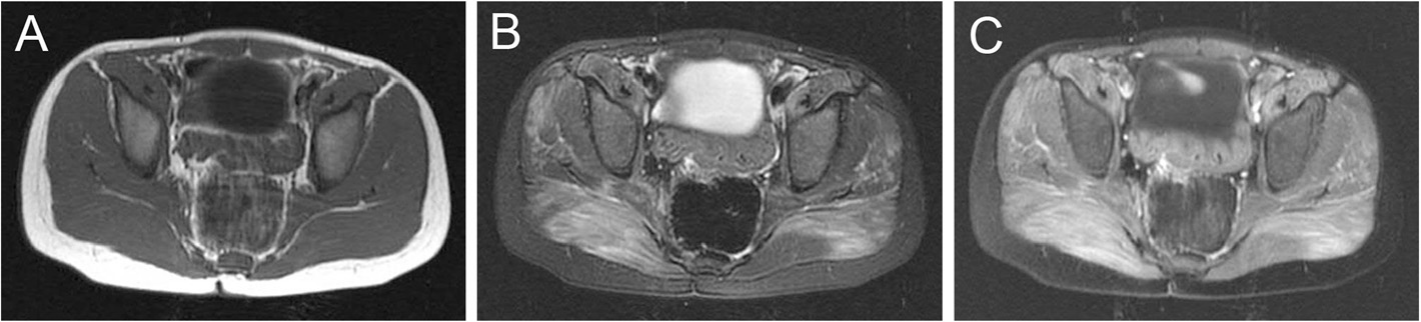
**MRI of the gluteus maximus muscles demonstrating enhancement consistent with myopathy.** (**A**) T1, (**B**) T2 fast spin echo (FSE), and (**C**) fat saturated T1 post gadolinium images demonstrate normal T1 signal and confluent increased T2 signal with associated gadolinium enhancement in the gluteus maximus muscles.

**Figure 2:**
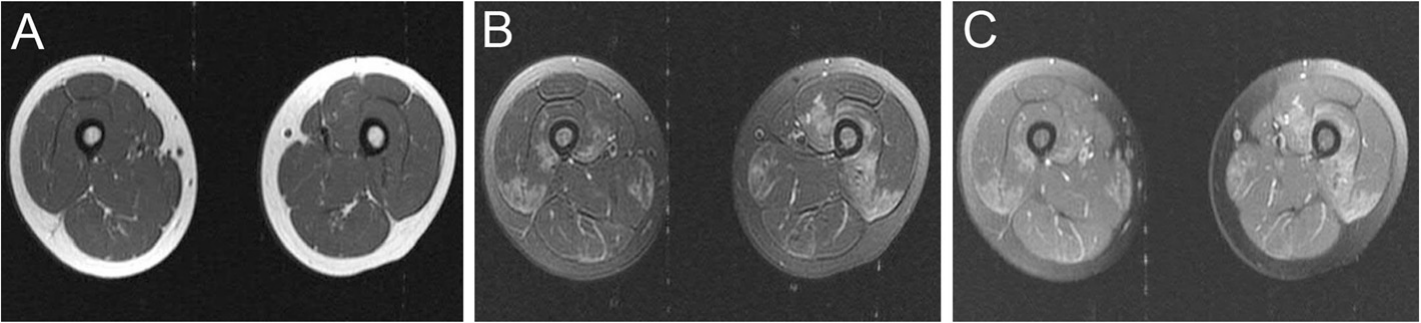
**MRI of the thigh musculature demonstrating enhancement consistent with myopathy.** (**A**) T1, (**B**) T2 FSE, and (**C**) fat saturated T1 post gadolinium images demonstrate normal T1 signal and patchy increased T2 signal with corresponding gadolinium enhancement in the vastus intermedius, vastus lateralis muscles.

Approximately one month later, the patient began to experience left knee pain without inciting injury. With the suspicion for lupus or JDM and new joint pain, the patient was referred to a pediatric rheumatologist. Physical exam demonstrated rash as noted previously, no arthritis of the knee though range of motion was tight, and no muscle weakness was found using manual muscle testing and Childhood Myositis Assessment Scale (CMAS). Serologic testing, including anti-nuclear antibody testing, inflammatory markers, and serum levels of muscle-derived enzymes were within normal limits. Given the negative ANA and physical exam findings; notably the distribution of the skin rash the presentation was thought to be more consistent with JDM than lupus erythematosus. An MRI was performed to evaluate for evidence of myositis. MRI showed patchy increased T2 signal with associated gadolinium contrast enhancement within the anterior, posterior, and medial compartment musculature of the thighs and within the gluteus maximus bilaterally, consistent with myopathy (Figures 1 and 2).

The combination of clinical, laboratory, and imaging findings were thought to be consistent with the diagnosis of JDM. The patient, however, did not satisfy complete diagnostic criteria for JDM; therefore muscle biopsy was recommended for confirmation and staging of the condition including inflammatory infiltrate amount and other changes to help aid in prognosis. Surgery was consulted for the muscle biopsy. Though the gluteus maximus appeared most diffusely involved on the MRI, the vastus lateralis is a more commonly and safely biopsied area. Due to the relative patchy involvement of the vastus lateralis, preoperative imaging localization was explored. Ultrasound of the gluteal and thigh muscles was performed, however the muscles demonstrated normal echotexture with no distinct abnormality to which the surgeons could be directed for biopsy. MRI guidance with wire localization for biopsy was requested.

**Figure 3:**
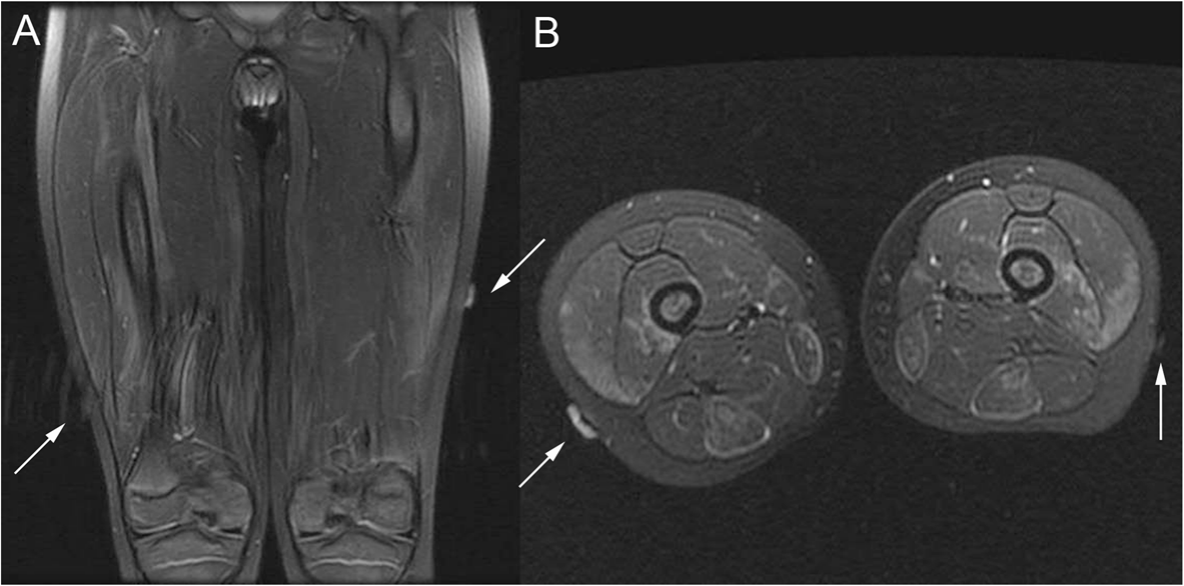
**MRI of skin fiducials on lateral thighs.** (**A**) Coronal and (**B**) axial T2 images show skin markers (arrows) overlying the high T2 signal within the vastus lateralis muscles bilaterally.

**Figure 4:**
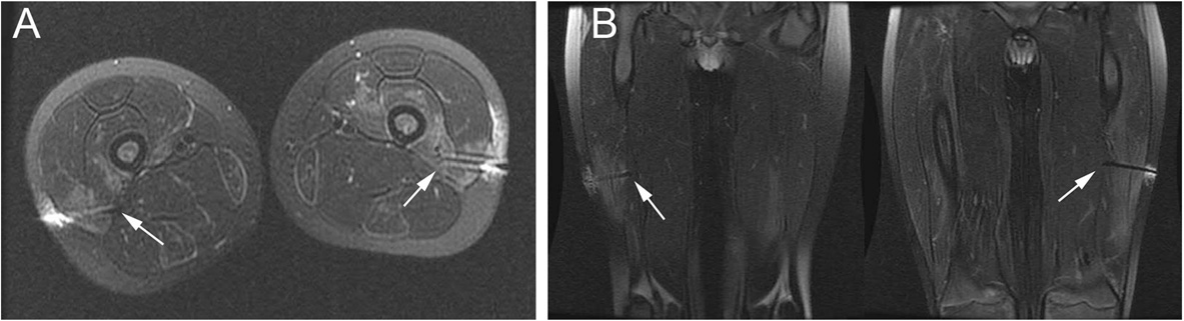
**MRI of the needle position within the high T2 signal in the vastus lateralis.** (**A**) Axial and (**B**) coronal T2 images performed post wire-localization show the localization wires (arrows) are located within regions of high T2 signal in the vastus lateralis muscles bilaterally.

The patient was brought to the MR (GE Signa 1.5 T, GE Medical, Milwaukee, WI) suite anteroom where anesthesia was initiated. The patient was transferred to the MR table in a supine feet-first position. Initial skin fiducials were placed on the lateral thighs bilaterally for reference markers (Figure 3). Coronal T2 images with fat saturation (TR 3000, TE 65, NEX 1, Flip angle 90, FOV 300, slice thickness 5 mm, echo train 8, matrix 256x224) were acquired to identify the position of the fiducials to the patchy muscular involvement in the vastus lateralis. Next axial T2 images with fat saturation (TR 4250, TE 66, NEX 2, Flip angle 90, FOV 280, slice thickness 5 mm, echo train 8, matrix 256×192) were acquired confirming entry position targeting the patchy involvement in both the right and left vastus lateralis. Using intermittent axial T2 images, 18-gauge MRI compatible wire localization needles were placed into the inferior aspects of each vastus lateralis at locations of increased T2 signal. Needle position within the high T2 signal in the vastus lateralis was confirmed with axial T2 images (Figure 4). The localizing wire was unsheathed into the muscle and the wires taped to the skin for surgical biopsy. Localization and placement of the localizing wires took approximately one hour.

The patient then proceeded to the operating room, where the localization wires led the surgeon directly to affected muscle tissue for biopsy. Evaluation of the biopsy specimen demonstrated perifascicular abnormalities and perivascular inflammation. Given the results of the skin and muscle biopsies, in addition to the clinical picture, the diagnosis was determined to be consistent with moderately severe active dermatomyositis. After pathologic confirmation of the JDM diagnosis, the patient was started on treatment with oral prednisone and methotrexate.

## Conclusions

Juvenile dermatomyositis is an autoimmune, inflammatory myopathy. Though a rare disease, it is the most common inflammatory myopathy in children with an incidence of 2–3 cases per million children per year [1,4]. Skin rashes and proximal muscle weakness are the most common symptoms, typically consisting of a heliotrope rash involving the eyelids, erythematous rash on the extensor surfaces (Gottron’s papules), and proximal muscle weakness [1,2]. Gastrointestinal, pulmonary, cardiac, and joint involvement, as well as other organ system involvement can also be seen, but is less common [5].

The diagnosis of JDM has historically been established using a combination of clinical, microscopic, laboratory, and electromyographic abnormalities. In 1975, Bohan and Peter published criteria for diagnosis of JDM [6]. Throughout the years, many additional authors have suggested adding to or modification of the Bohan and Peter criteria for diagnosis of JDM. For example, Tanimoto *et al.*[7] suggested the addition of more clinical findings to the diagnostic criteria, while Love *et al.*[8] suggested diagnostic classification based upon presence of specific autoantibodies.

Imaging findings are not included in the criteria for diagnosis of JDM; however, many clinicians have been using imaging as a tool for diagnosis and monitoring of disease activity [2]. Ultrasound may demonstrate edema and swollen muscle fibers in the affected muscles [9]. MRI findings of symmetric muscular edema are typical of JDM, however the intramuscular edema may be patchy [2,10,11]. Fatty infiltration and muscle atrophy may be evident on T1 MRI images in chronically affected muscles [2,3,10].

Prior to the availability of immunosuppressive medications, the prognosis of patients with JDM was poor. While approximately one-third of these patients recovered without sequelae of their disease, another one-third of these patients died from their disease, and the remaining one-third survived but had serious permanent disability [12]. The availability of corticosteroid medications significantly decreased the mortality rate. Current treatment of JDM typically consists of oral or IV corticosteroids, often in combination with other immunosuppressive drugs, most commonly methotrexate. With the currently available treatments, the mortality rate is now estimated to be one to two percent [13]. Given the dramatically decreased mortality rates in patients treated with immunosuppressive therapy, prompt diagnosis and treatment of JDM is imperative.

For cases in which the diagnosis of JDM is unclear or the clinician seeks to stage the severity of the condition in efforts to predict clinical course and guide therapy regimens, the radiologist may be asked to assist by evaluating for imaging evidence of myopathy or to perform biopsy or localization for surgical excisional biopsy. Studies by Crowe *et al.*[14], Wargula *et al.*[15], and Miles *et al.*[16] have supported the predictive value of muscle biopsy in the treatment and prognosis of JDM.

Preoperative hookwire localization has long been performed in breast imaging, typically using mammographic and/or ultrasound guidance. More recently, with the advent of MRI compatible equipment, hookwire and needle localization has also been performed in breast imaging using MR guidance [17]. A multi-center trial examined MRI guided wire localization of clinically and mammographically occult lesions of the breast and found it to be beneficial, especially in lesions that were difficult to see mammographically [18]. Preoperative wire localization has expanded to be used to localize lesions in multiple locations throughout the body, including intramuscular lesions [19].

Our case illustrates that MRI guided wire localization is a feasible technique for targeting affected muscle for surgical excisional biopsy in cases where there is patchy distribution of pathology seen only with MRI. Future investigations will seek to evaluate this methodology in assisting the work up of patients with difficult or confusing clinical presentations of JDM.

## Consent

The institutional review board (IRB) at our institution was contacted regarding this case; however it was determined that IRB approval, consenting, and/or a protocol were not required for this single retrospective case report. All data was handled confidentially with no identifying information.

## Abbreviations

JDM: Juvenile dermatomyositis; CMAS: Childhood myositis assessment scale; FSE: Fast spin echo; IRB: Institutional review board.

## Competing interests

The authors declare that they have no competing interests.

## Authors’ contributions

VT drafted the manuscript and revised it critically for important intellectual content. SZ participated in the study design and coordination and helped draft the manuscript. CM performed the surgical muscle biopsy. AR conceived the study, participated in the study design and coordination, and has given final approval of the version to be published. JM participated in the study and the design of the procedure. DW participated in the study design, performed the MRI with guide wire localization, revised the manuscript critically for important intellectual content, and has given final approval of the version to be published. All authors read and approved the final manuscript.
